# Three-dimensional visualization and navigation for micro-noninvasive uterine fibroid surgery based on MRI and ultrasound image fusion

**DOI:** 10.3389/frai.2025.1613960

**Published:** 2025-07-23

**Authors:** Ting Wang, Yingang Wen, Zhibiao Wang, Xi Li

**Affiliations:** ^1^Foundation Department, Chongqing Medical and Pharmaceutical College, Chongqing, China; ^2^National Engineering Research Center of Ultrasonic Medicine, Chongqing, China; ^3^State Key Laboratory of Ultrasound in Medicine and Engineering, College of Biomedical Engineering, Chongqing Medical University, Chongqing, China

**Keywords:** uterine fibroids, focused ultrasound ablation, MRI-ultrasound image fusion, surgical navigation, real-time ultrasound tracking

## Abstract

**Objective:**

To address the challenges of low surgical precision and poor consistency in focused ultrasound ablation surgery (FUAS) for uterine fibroids, which are often caused by variations in clinical experience and operator fatigue, this study aims to develop an intelligent three-dimensional (3D) visualization and navigation system by integrating magnetic resonance imaging (MRI) with real-time ultrasound (US) imaging, thereby improving the accuracy and efficiency of uterine fibroid surgery.

**Methods:**

MRI and US images from 638 patients were annotated by experienced clinicians. The nnU-Net algorithm was used for preoperative segmentation and 3D reconstruction of MRI images to provide detailed visualization of fibroid morphology. The YOLACT model was applied to achieve rapid delineation of the uterus and key anatomical structures in real-time US images. To enhance the accuracy of lesion localization and navigation, the Iterative Closest Point (ICP) algorithm was employed for the registration of preoperative MRI with intraoperative US images.

**Results and discussion:**

Experimental results demonstrated that the system achieved a Dice Similarity Coefficient (DSC) exceeding 90% for the segmentation and identification of anatomical structures such as the uterus and fibroids. The YOLACT model achieved an accuracy greater than 95% in identifying key structures in real-time US images. In 90% of the cases, the system enabled efficient and precise tracking; however, approximately 5% of the cases required manual adjustment due to discrepancies between patient anatomy and preoperative MRI data. The proposed intelligent navigation system, based on MRI–US image fusion, offers an efficient and automated solution for FUAS in treating uterine fibroids, significantly improving surgical precision and operational efficiency. This system demonstrates strong clinical applicability. Future research will focus on enhancing the adaptability of the system, particularly in addressing challenges such as significant tissue deformation and occlusion, to improve its robustness and applicability in complex clinical scenarios.

## 1 Introduction

**Figure 1:**
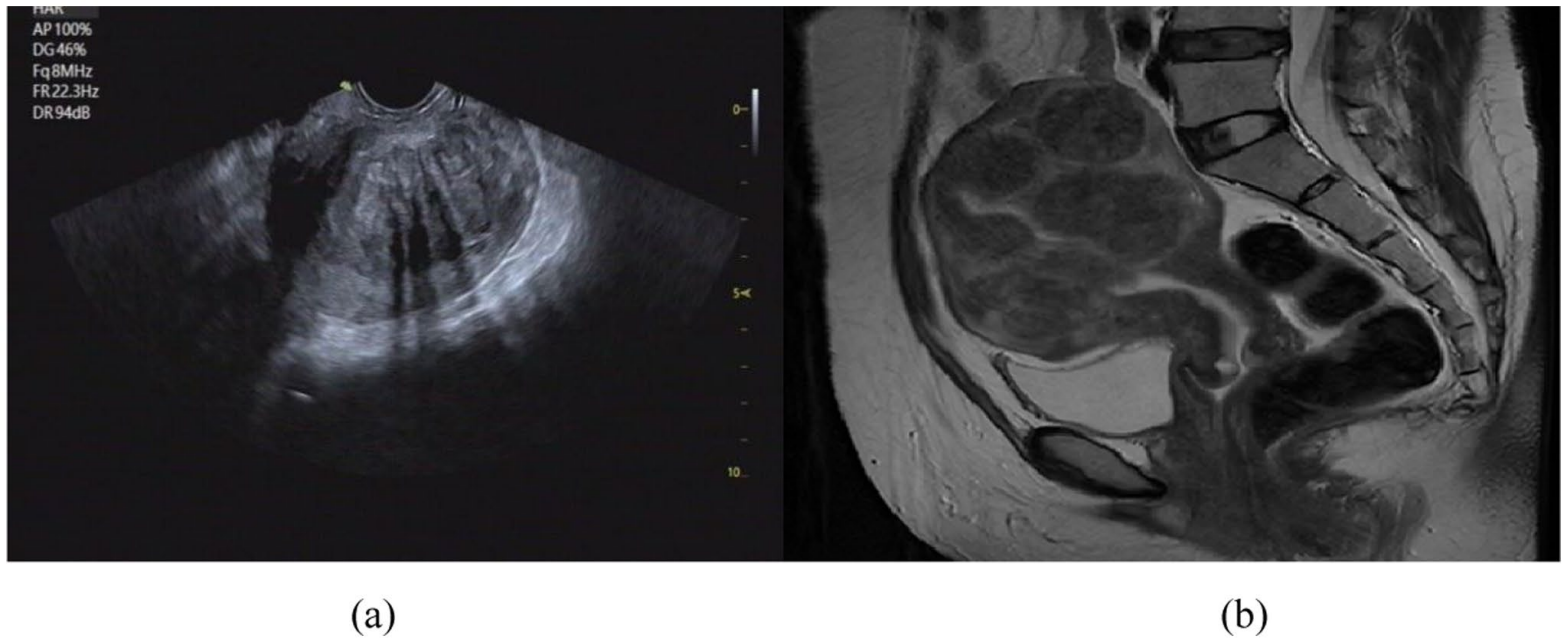
Comparison of imaging effect between US image **(a)** and MRI image **(b)**. **(a)** US image of uterine fibroids; **(b)** MR image of uterine fibroids.

Uterine fibroids are among the most prevalent benign tumors of the female reproductive system. In recent years, their incidence has steadily increased, posing a significant threat to the physical and psychological well-being of women of reproductive age (Sparic et al., 2016; Okolo, 2008). FUAS has emerged as a widely adopted therapeutic approach due to its non-invasive nature, absence of ionizing radiation, and ability to preserve uterine structure and function (Wang et al., 1997; Ziglioli et al., 2020; Kashyap and Kashyap, 2001). Ultrasound and MRI are two complementary imaging modalities that, when integrated, provide synergistic anatomical and functional information (Ries et al., 2010; Lovegrove, 2006). Ultrasound offers real-time imaging, low cost, portability, and widespread clinical accessibility, making it well-suited for intraoperative guidance. In contrast, MRI provides high-resolution, three-dimensional soft-tissue contrast, which is critical for preoperative planning but lacks real-time imaging capabilities (Zhao et al., 2013; Wang et al., 2024). The differences in uterine fibroid visualization between MRI and US are illustrated in Figure 1.

For accurate identification and registration of uterine fibroid regions in ultrasound and MR images, Luo et al. (2021) proposed a method for automatic segmentation and registration of uterine fibroid ultrasound images. First, ReFineNet was used to segment the complete fibroid contour in handheld ultrasound images, and the upper boundary of the fibroid in the guidance ultrasound image was manually annotated. Then, the two ultrasound images were registered. Zhang et al. (2023) proposed an attention-based deep learning method for automatic segmentation of uterine fibroids in preoperative MR images. The proposed method is based on a U-Net architecture, incorporating channel attention through squeeze-and-excitation blocks with residual connections, as well as spatial attention via a pyramid pooling module. This method achieved good segmentation results but is limited to two-dimensional images. Boneš et al. (2024) developed an automated system for uterine shape segmentation and alignment based on 3D ultrasound data. First, deep learning techniques were used to automatically segment the uterus in 3D ultrasound scans. Then, standard geometric methods were applied to align the segmented shapes, enabling the extraction of the normal uterine shape for further analysis.

During FUAS procedures, real-time ultrasound plays a pivotal role in intraoperative navigation. However, its relatively low spatial resolution and susceptibility to acoustic shadowing and reflection artifacts often hinder the precise delineation of fibroid boundaries. Conversely, preoperative MRI provides clear, three-dimensional anatomical visualization of the uterus and fibroids, allowing accurate assessment of their morphology, size, and spatial distribution. Integrating preoperative MRI with intraoperative US through image registration and fusion techniques enhances the intraoperative visibility of fibroid margins in real-time, thereby improving surgical accuracy and procedural efficiency. To meet these clinical demands, we have developed a 3D visualization model based on MRI–US image fusion. This model supports the entire FUAS treatment workflow, including preoperative planning, intraoperative navigation, and postoperative evaluation. It allows surgeons to anticipate anatomical challenges, assess procedural risks, and manage potential complications more effectively, thus reducing intraoperative uncertainty and enhancing surgical success rates and patient safety. Moreover, 3D visualization plays a crucial role in postoperative follow-up by enabling comprehensive comparisons between pre- and post-operative anatomical structures. This capability improves the assessment of ablation efficacy, facilitates lesion monitoring, and supports early detection of recurrence, contributing to long-term treatment optimization and patient management.

In 2019, Chongqing Haifu Medical Technology Co., Ltd. introduced a prototype MRI–US fusion and navigation system. However, its clinical application has been limited by several technical challenges. Physiological changes during surgery and spatiotemporal discrepancies between preoperative MRI and intraoperative US often necessitate repeated manual registration, significantly increasing the clinical workload and reducing procedural efficiency. Thus, the development of intelligent algorithms capable of real-time, automated image registration and tracking is crucial for improving clinical usability. Nevertheless, achieving accurate MRI–US registration in the context of uterine fibroid treatment remains a formidable challenge due to several factors: differences between imaging modalities, non-rigid tissue deformation during surgery, and poorly defined fibroid boundaries. These challenges are further compounded by the need for real-time performance and the differing demands of radiological diagnosis versus surgical navigation. Consequently, the development of a robust, intelligent, and clinically adaptable navigation system remains a critical research objective in the field of medical image processing.

Conventional MRI segmentation and ultrasound image interpretation techniques exhibit significant limitations in FUAS applications. Their inability to accommodate the dynamic nature of the intraoperative environment and lack of real-time tracking and registration capabilities severely restrict their utility in surgical planning and navigation. To address these challenges, this study proposes a comprehensive, integrated solution that incorporates MRI segmentation, ultrasound image analysis, multimodal image fusion, and real-time lesion tracking. By closely aligning system development with clinical requirements, we aim to create a fully automated MRI–US image registration and tracking system specifically designed for real-time guidance during FUAS procedures. Ultimately, this system seeks to significantly improve the precision, safety, and operational efficiency of minimally invasive FUAS treatment for uterine fibroids.

## 2 Methods

### 2.1 Patients

A database of 638 patients diagnosed with UF at Chongqing Haifu Hospital from January 2021 to January 2023 was collected for this study. This research was conducted with the approval of the hospital’s ethics committee and had no implication on patient treatment.

### 2.2 Image segmentation

#### 2.2.1 MR image segmentation

**Figure 2:**
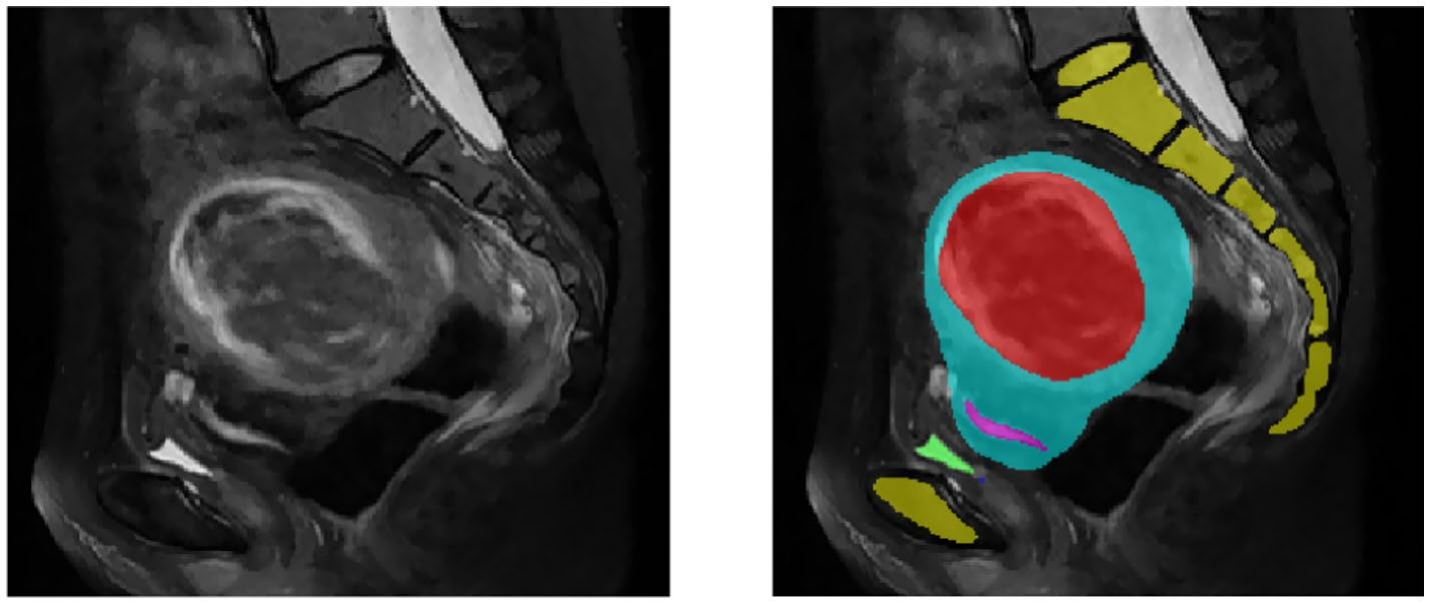
MR images of uterine fibroids. The yellow area is the spine, the red area is the fibroid, the blue area is the uterus, the pink area is the endometrium, and the green area is the bladder.

Segmentation of MR images of uterine fibroids plays a critical role in medical image analysis, directly influencing the accuracy and effectiveness of subsequent image registration and fusion. Traditionally, manual segmentation by clinicians is time-consuming, prone to low accuracy, and often lacks consistency, thus limiting its clinical applicability. Therefore, the development of automated or semi-automated segmentation methods has become imperative. MR images of uterine fibroids present numerous challenges due to their high resolution, complex anatomical structures, relatively small lesion areas, ambiguous boundaries, diverse textural features, anisotropy, and irregular shapes, as illustrated in Figure 2. Conventional segmentation techniques such as level-set methods, region growing, and active contour (snake) models have been previously applied to uterine MRI image analysis (Yao et al., 2006). However, these approaches fall short in terms of precision and efficiency, making them unsuitable for clinical implementation. With the advent and continuous advancement of deep learning technologies, medical image segmentation has seen groundbreaking progress. Nonetheless, their application in the context of uterine fibroid treatment remains insufficiently validated. While deep learning methods have achieved promising results across various segmentation tasks, challenges such as small sample sizes and heterogeneous data still hinder their widespread adoption in clinical environments. This study aims to establish a reliable and efficient segmentation framework that meets clinical demands and improves therapeutic outcomes.

Our research team has explored several deep learning models, including V-Net (Milletari et al., 2016) and U-Net (Tang et al., 2020; Norman et al., 2018), and has collaborated with Southeast University to co-develop the HIFUNET model (Zhang et al., 2020). These efforts have led to significant improvements in segmentation accuracy, with precision rates exceeding 85%, outperforming traditional algorithms. However, these results still fall short of meeting the stringent accuracy requirements of clinical practice.

The nnU-Net (Isensee et al., 2018) architecture, based on the classical U-Net structure, is an adaptive convolutional neural network framework that incorporates an advanced, automated pipeline for medical image segmentation. The combination of both architectures offers strong complementarity in model integration. Owing to its simplicity, flexibility, and adaptability to diverse biomedical image datasets, nnU-Net was selected as the segmentation network for this study.

**Figure 3:**
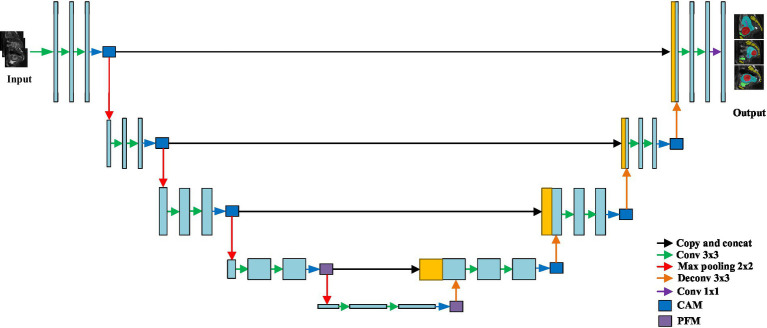
Network architecture.

**Figure 4:**
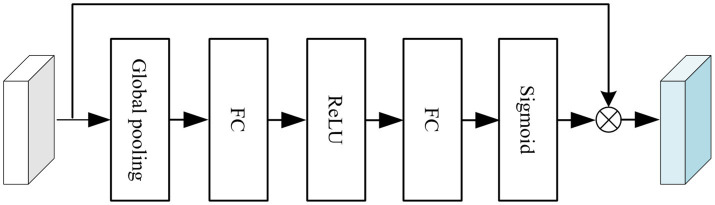
Channel attention module (CAM).

To enhance segmentation accuracy, a channel attention module (CAM) and a pyramid fusion module(PFM) were integrated into the original nnU-Net architecture, as illustrated in Figure 3. The structure of the CAM is shown in Figure 4. Specifically, global average pooling is first applied to the input feature map to obtain a channel-wise global descriptor. This descriptor is then passed through two fully connected (FC) layers with a ReLU activation in between to capture the inter-channel dependencies. Subsequently, a Sigmoid activation function is used to generate attention weights for each channel. These weights are multiplied with the original feature map in a channel-wise manner to enhance informative features and suppress irrelevant or redundant ones, thereby improving the model’s sensitivity to key regions. The CAM effectively models channel-wise dependencies and adaptively adjusts the importance of each feature channel, enabling the network to emphasize features that are more relevant to the segmentation task while reducing the influence of less important information. This enhances the representational capability of the model, improves the recognition of target regions and boundaries, and contributes to higher segmentation accuracy.

**Figure 5:**
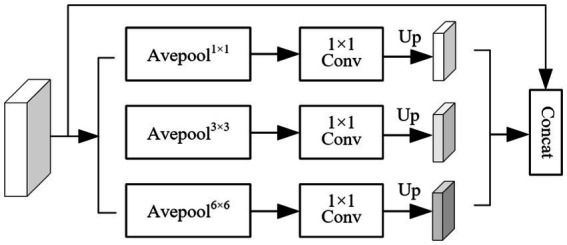
Pyramid fusion module (PFM).

The structure of the pyramid fusion module (PFM) is shown in Figure 5. It first extracts multi-scale information from the input feature map using average pooling operations with three different kernel sizes, enabling the capture of local features at various levels. Next, Con1 × 1 is applied to the pooled feature maps, followed by upsampling through bilinear interpolation to restore the spatial resolution to match that of the original feature map. Finally, features from different scales are concatenated to form the output of the PFM. The PFM effectively extracts and integrates multi-scale features, enhancing the model’s ability to perceive objects of varying sizes. By performing pooling, convolution, and fusion at multiple spatial scales, the module captures both fine-grained details and global contextual information, thereby improving the model’s capability to recognize object boundaries and complex structures. This leads to improved segmentation accuracy and enhanced robustness in diverse scenarios.

nnU-Net employs a two-stage training strategy to optimize model performance. In the first stage, the model is trained on downsampled images to obtain preliminary segmentation results. In the second stage, these initial segmentation outputs are upsampled to the original voxel spacing and used as inputs for further training under full-resolution conditions. This approach not only enhances the model’s ability to capture fine image details but also improves segmentation accuracy when handling large-scale or complex structural data. The design of nnU-Net enables automatic adjustment of network parameters across different layers to ensure no spatial information is lost when processing images of varying resolutions. Specifically, the network architecture is first adapted according to the input image size to optimize processing efficiency. Subsequently, the number of convolutional layers and kernel sizes are selected based on data characteristics to optimize feature extraction. The positions and quantities of pooling and upsampling layers are then adjusted to preserve image resolution and information integrity. Finally, depending on the task complexity and available computational resources, the number of channels in each layer is tuned to balance computational cost and model performance.

The total loss function is defined in Equation 1:

(1) $$L_{\text{tota}l}=L_{\text{dice}}+L_{\mathrm{CE}}$$

Where, *L_dice_* and *L_CE_* are shown in Equations 3 and 4:

(2) $$L_{\text{dice}}=-\frac{2}{|K|}\sum_{k\in K}\frac{\sum_{i\in I}u_{i}^{k}v_{i}^{k}}{\sum_{i\in I}u_{i}^{k}+\sum_{i\in I}v_{i}^{k}}$$

(3) $$L_{\mathrm{CE}}=-\frac{1}{N}\sum_{i=1}^{N}[v_{i}\ln(u_{i})+(1-v_{i})\ln(1-u_{i})]$$

Where, *u* is the softmax output of the network, and *v* is the true label of the segmentation map. The shapes of *u* and *v* are both *N* × *K*, where *i* ∈ *N* represents the number of pixels in the training block/batch, and *k* ∈ *K* represents the number of categories.

#### 2.2.2 US image segmentation

Real-time US images of uterine fibroids exhibit several unique characteristics, including:

1. Low signal-to-noise ratio: real-time ultrasound images are highly susceptible to speckle noise, which compromises image clarity and hinders accurate analysis.
2. Grayscale inhomogeneity: the internal tissues of uterine fibroids often display significant grayscale variability, making consistent segmentation challenging.
3. Distinct membrane boundaries: the surface of uterine fibroids is typically enclosed by a membranous structure with markedly different acoustic impedance from the underlying tissues, resulting in strong interface reflections and a relatively clear contour. However, due to lesion-specific influences, the boundaries—especially the deeper ones—may appear blurred and difficult to discern.

**Figure 6:**
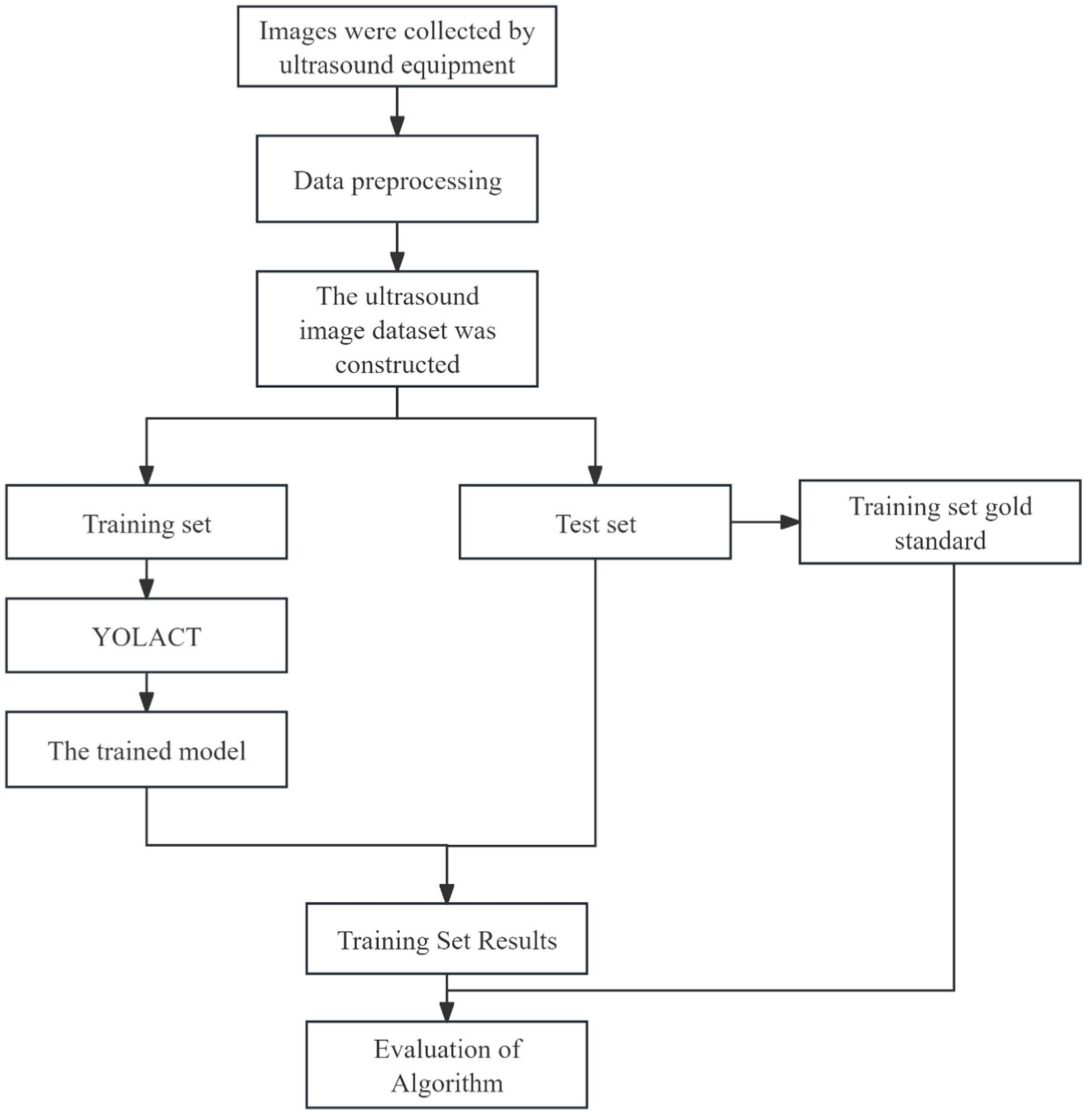
The process of uterine fibroid segmentation in ultrasound images.

In the current ultrasound image segmentation network, Mask R-CNN (He et al., 2017) achieves a processing speed of approximately 10 frames per second (FPS), whereas YOLACT (Bolya et al., 2019) can reach 30 FPS while maintaining the same level of accuracy. Given the real-time performance requirements of clinical applications, both speed and accuracy are essential. Therefore, YOLACT was selected as the ultrasound image segmentation network for subsequent research. The parameters of the YOLACT model were first adjusted to construct an automatic segmentation model specifically tailored to the uterine fibroid region, enabling efficient and automated processing of ultrasound images. The model was then applied to analyze dynamic changes in ultrasound images in real time, accurately segmenting the target region and seamlessly integrating the results into the FUAS 3D-assisted imaging system. This enabled real-time tracking and visualization of tissue changes. The segmentation workflow is illustrated in Figure 6. To further improve the efficiency and adaptability of the YOLACT approach—particularly to achieve real-time performance on devices with lower computational power—the image size was reduced to half of its original dimensions while preserving essential image information. This optimization ensured that real-time processing requirements could also be met on standard computing systems.

### 2.3 Registration algorithm

Three-dimensional point cloud registration is a core component of the 3D reconstruction process. Its objective is to align point cloud data obtained from multiple perspectives and various reference coordinate systems into a unified coordinate system. This alignment is achieved through precise rotational and translational transformations, thereby capturing complete object information and laying the technical foundation for subsequent visualization and analysis. Among point cloud registration algorithms, the Iterative Closest Point (ICP) algorithm is widely adopted due to its high efficiency and accuracy. ICP refines registration results iteratively and typically achieves higher precision compared to traditional methods. However, since it relies primarily on Euclidean distance to compute nearest-point correspondences, its performance may be limited when applied to deformable biological tissues such as soft organs. To further enhance the performance of ICP in this study, we implemented targeted optimizations that comprehensively account for the ICP characteristics of key anatomical structures, the dynamic behavior of surrounding tissues, and the correlation between motion and deformation. These optimizations improve registration accuracy for complex, deformable biological structures and ensure the reliability and precision of 3D reconstruction. Accurate representation of anatomical structures is fundamental for effective treatment and is particularly critical for preoperative planning and intraoperative navigation.

**Figure 7:**
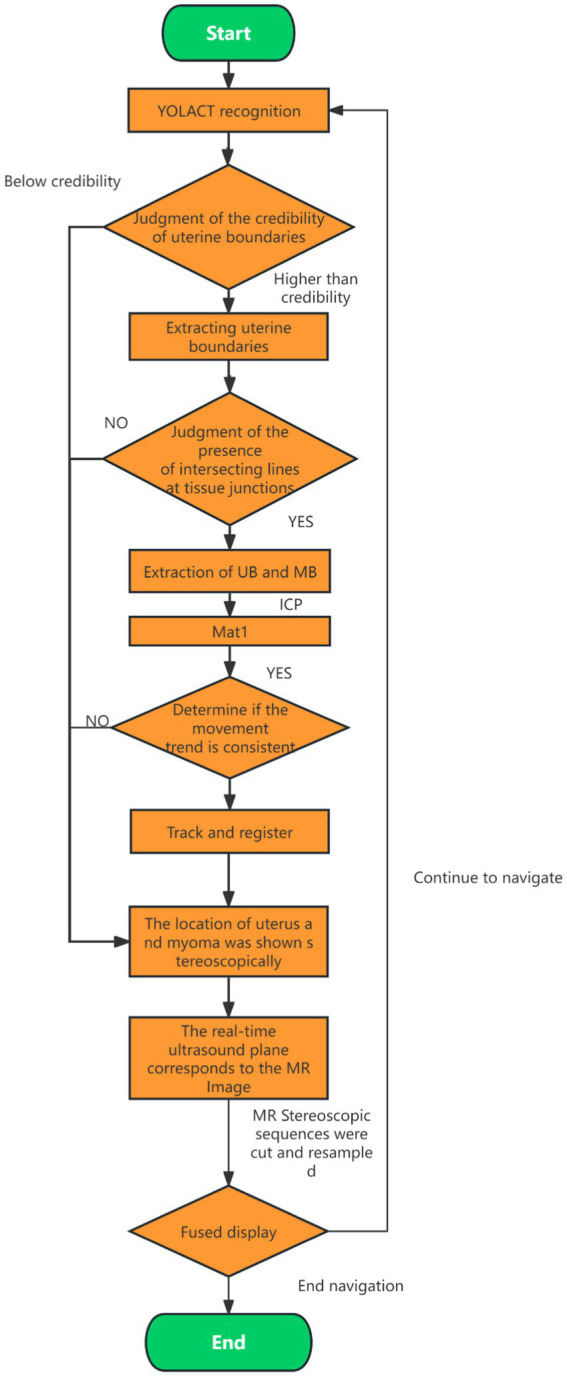
Flow chart of ICP algorithm registration.

The core of the ICP algorithm lies in establishing initial correspondences between two point clouds, which directly impacts the number of iterations, runtime, and registration accuracy. An ideal initial correspondence significantly reduces iteration count, shortens computation time, and enhances precision. In this study, we selected the uterus as the primary target for ICP registration to fully leverage its anatomical stability and maximize registration performance. The process begins by applying the YOLACT model to real-time ultrasound images to accurately identify uterine boundaries, especially those near the abdominal wall. The ICP algorithm is then applied to refine the initial correspondences established by YOLACT and optimize registration through iterative processing. The main workflow is illustrated in Figure 7. By combining YOLACT-based boundary detection with ICP-based precision registration, we significantly improved the accuracy of uterine segmentation and alignment in real-time ultrasound images, thereby ensuring the robustness and fidelity of the 3D reconstruction. This workflow satisfies the stringent requirements of FUAS surgical navigation.

The specific registration steps are as follows:

1. YOLACT is used to detect the boundaries of the uterus, skin, and bladder in real-time ultrasound images. Uterine boundary reliability is assessed, and any boundary data falling below a predefined confidence threshold is excluded.
2. The uterine boundary is extracted and validated by checking for intersection lines at junctions with the bladder and skin. Boundaries without valid intersections are excluded from further tracking.
3. The lower half of the uterine boundary is extracted from both the ultrasound and MRI images. The ICP algorithm is applied to these boundaries to compute a transformation matrix (Mat1). If Mat1 deviates from the expected probe motion trend, it is excluded from the registration and tracking process.
4. Using Mat1, spatial transformation is performed on the reconstructed MRI volume to determine the positions of the uterus and tumor within the treatment field. The MRI volume is sliced and resampled to match the real-time ultrasound image sequence.
5. MRI and real-time ultrasound images are fused for display. Tumor boundaries extracted from MRI are overlaid onto the real-time ultrasound images to guide treatment.

**Figure 8:**
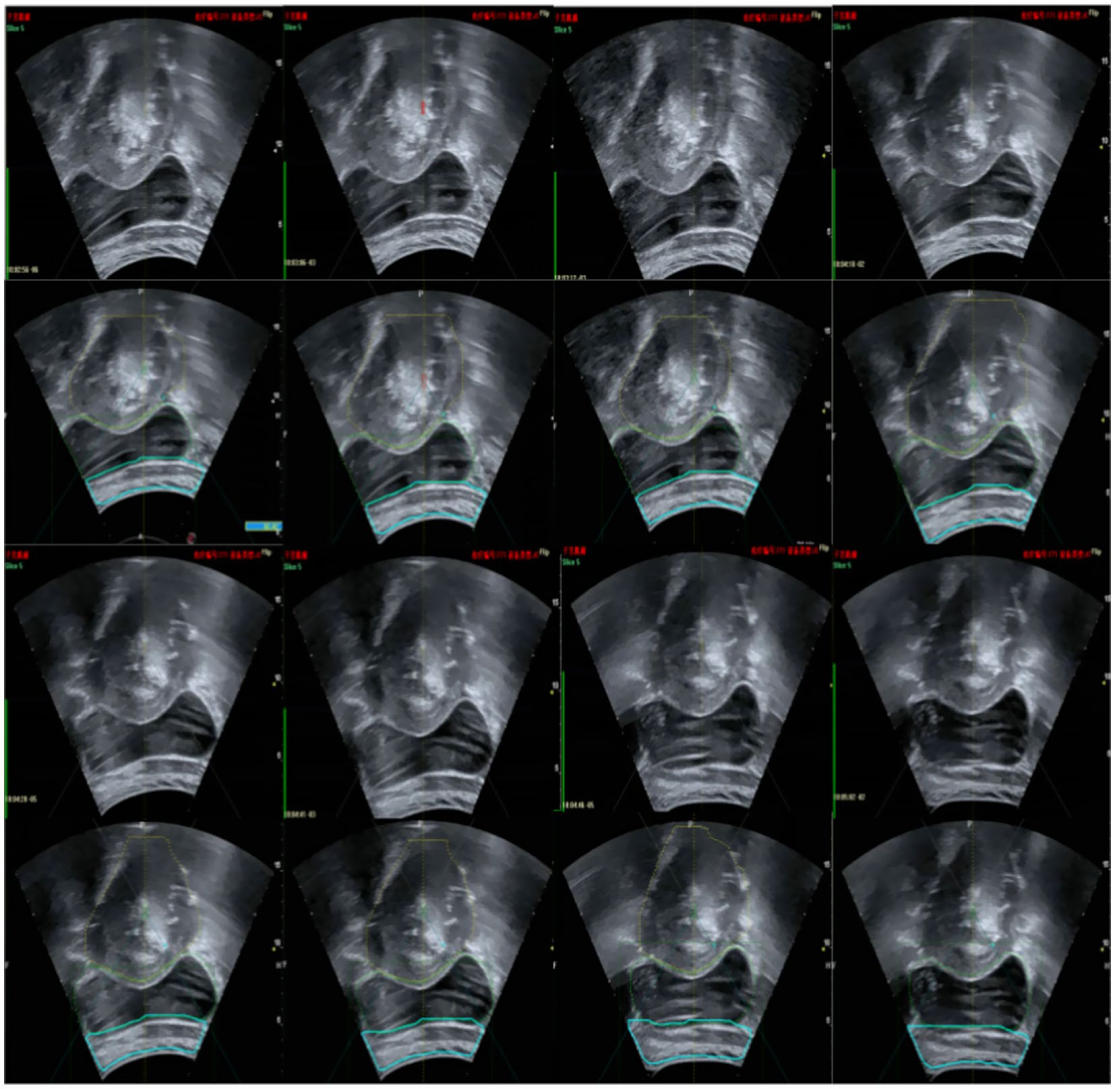
Ultrasonic video stream tracking image.

Following the fusion process, tumor boundaries from MRI are accurately superimposed on the real-time ultrasound images as needed, providing precise treatment guidance and ensuring both safety and efficiency during FUAS procedures. The real-time intraoperative tracking results are shown in Figure 8. These results demonstrate that the tracking system can maintain synchronization with the target even under conditions involving tissue deformation and displacement caused by probe movement.

### 2.4 3D visualization

**Figure 9:**
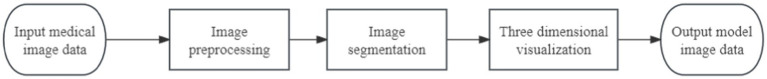
Visualization steps of 3D reconstruction of medical images.

**Figure 10:**
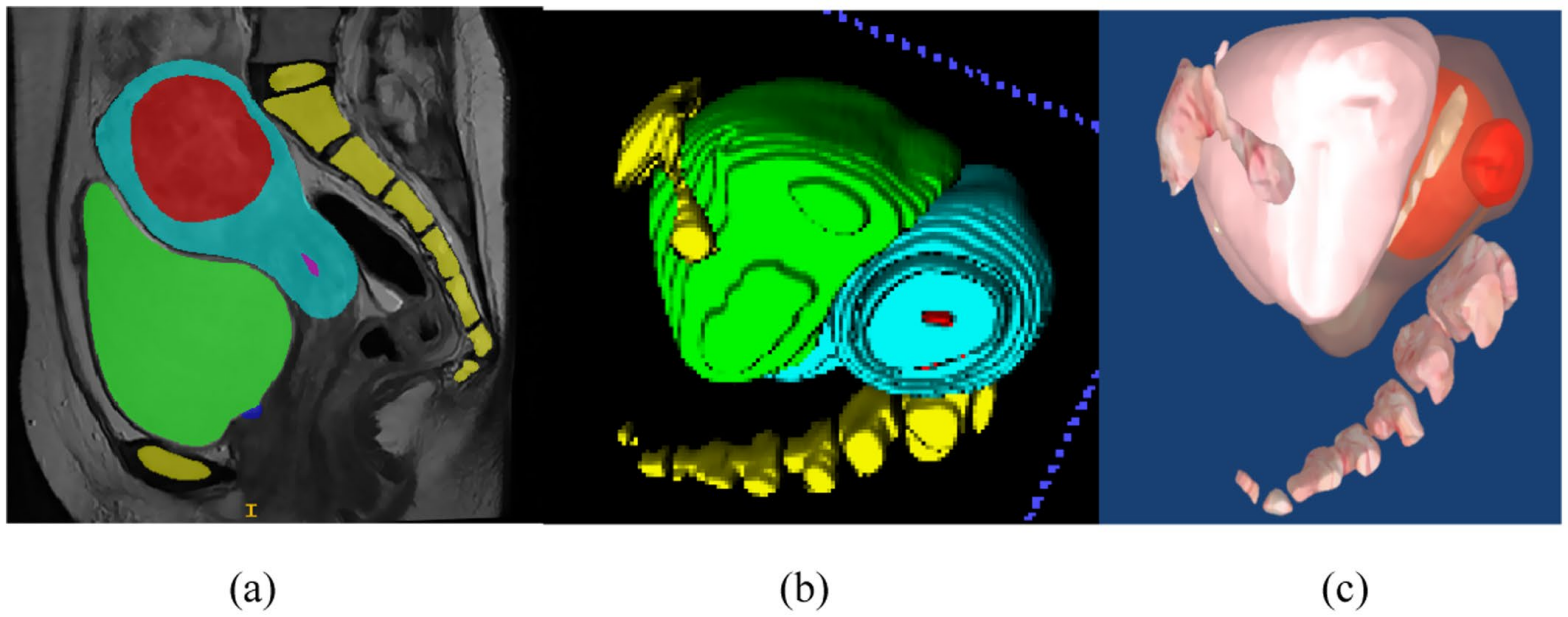
3D visualization process. **(a)** Schematic representation of uterine fibroid segmentation, **(b)** reconstructing 3D models, **(c)** the 3D model was reconstructed and smoothed.

3D visualization refers to the process of converting two-dimensional medical image sequences into 3D models, as illustrated in Figure 9. As shown in Figure 10a, once the surface structures of various organs are accurately segmented, the anatomical boundaries can be determined and subsequently applied to a 3D surgical navigation system. By integrating automatic recognition techniques, the system achieves 3D surface reconstruction of the segmented results, as demonstrated in Figure 10b. For MRI, the system can accurately identify anatomical structures such as the uterus, fibroids, and bladder, and perform corresponding 3D reconstructions. From Figure 10b, it is evident that the initial surface reconstruction yields a satisfactory level of visual presentation. To further enhance the visualization quality of FUAS procedures, surface smoothing techniques were applied to eliminate the stair-step artifacts. In addition, texture mapping methods were employed to optimize the 3D representation, as shown in Figure 10c.

**Figure 11:**
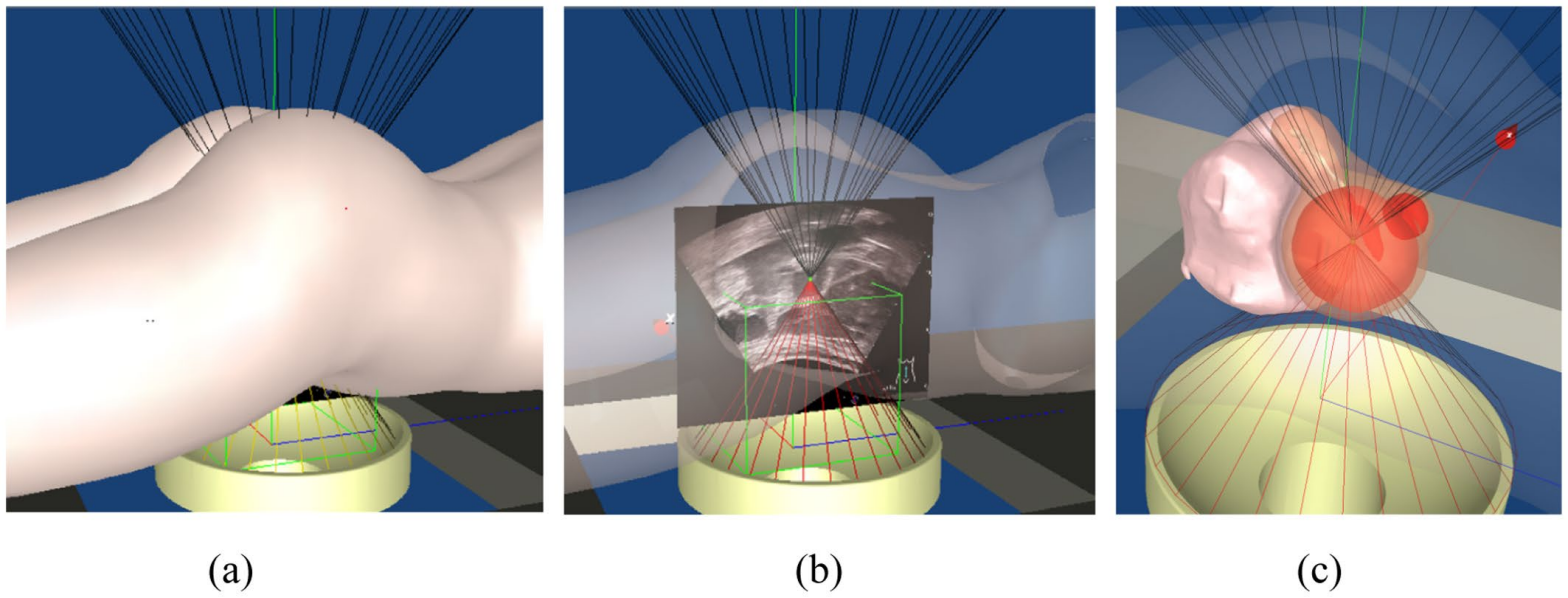
3D surgical navigation system. **(a)** Opaque, **(b)** 50% transparency, **(c)** local magnification of uterine fibroid.

**Figure 12:**
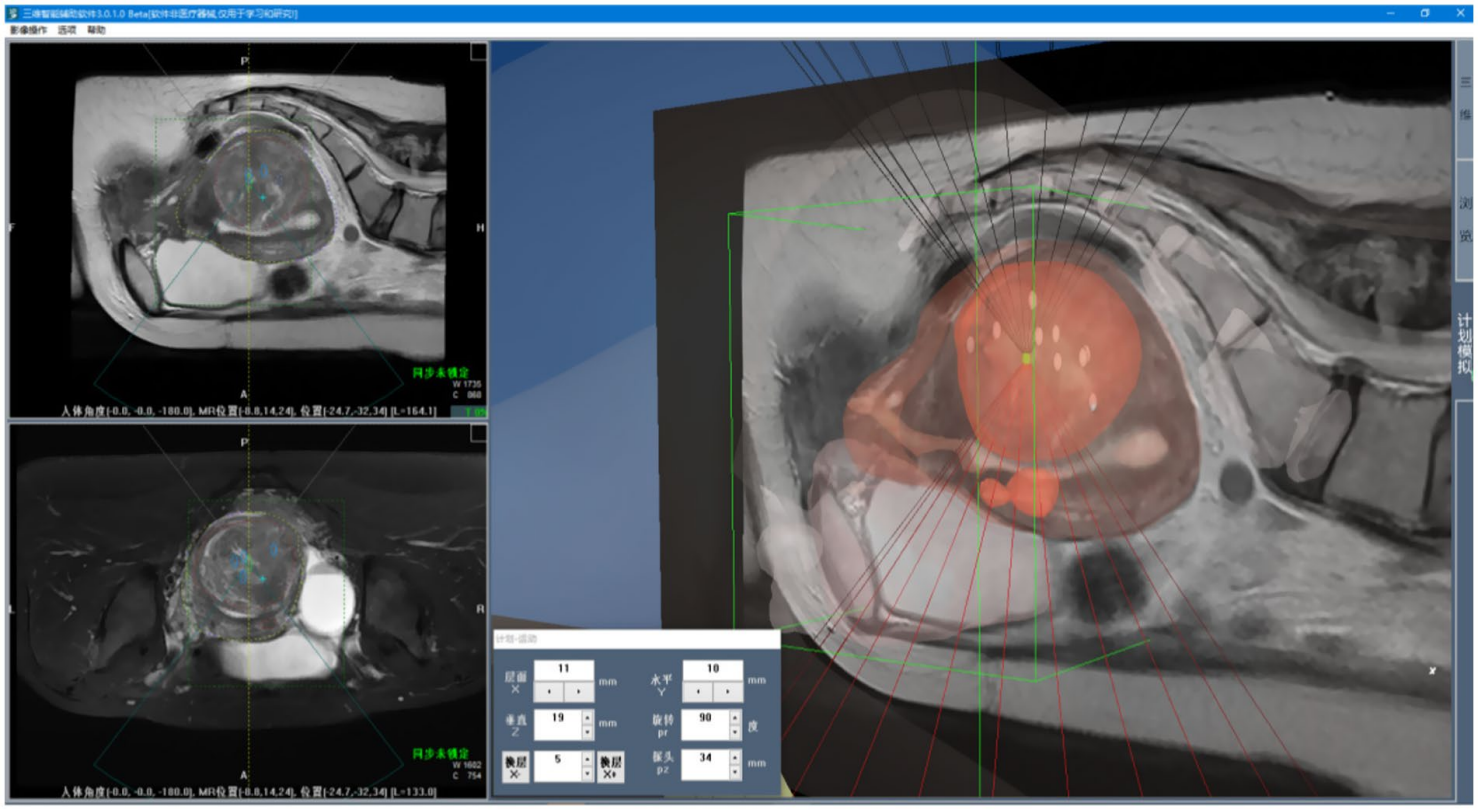
3D plan simulation module.

Given the large number of anatomical structures involved and the potential for visual occlusion, we optimized the visualization modes based on clinical usage patterns and physician preferences. A set of innovative animated display modes was designed, including Organ Reconstruction Mode, Treatment Scene Mode, and Irradiation Mode, as shown in Figure 11. These modes support automatic display and adjustable transparency, allowing specific tissues and structures to be selectively hidden as needed. They also enable automatic viewpoint adjustment to accommodate the visualization requirements of various clinical scenarios. Furthermore, a 3D Preoperative Planning Simulation Module was developed to enable surgeons to meticulously plan and simulate the entire surgical process prior to the actual operation. This functionality significantly enhances both the precision and safety of FUAS interventions. The effectiveness of this feature is illustrated in Figure 12. The implementation of this visualization framework also lays a solid technical foundation for subsequent MRI–ultrasound image fusion tasks.

### 2.5 Trend analysis module

**Figure 13:**
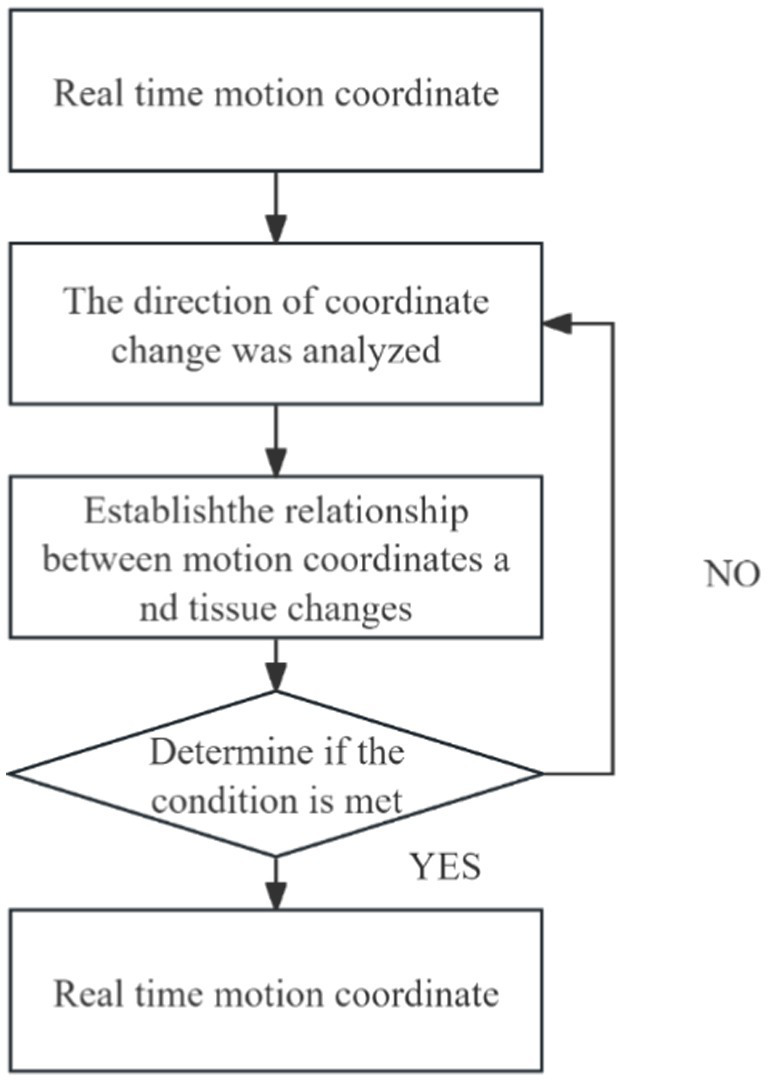
Flow chart of probe movement trend analysis.

In clinical practice, changes in patient posture can lead to significant organ deformation, making it difficult to automatically register and track intraoperative ultrasound images with preoperative MR images. As a result, clinicians are often required to perform manual adjustments and rely on conventional analytical methods for comprehensive assessment. To address this challenge, we developed a Trend Analysis Module to eliminate erroneous data that deviate from expected patterns during image registration and tracking. This module conducts trend analysis based on the anticipated motion and deformation patterns of anatomical structures, enabling the identification and exclusion of data points that fall outside the acceptable range. The detailed workflow is illustrated in Figure 13.

With the integration of this module, the system’s false recognition rate was effectively reduced, and the accuracy and robustness of tracking were significantly improved. The combination of automated registration and tracking with clinician-guided decision-making facilitates the construction of a more reliable and precise intraoperative guidance system. This contributes to improved patient outcomes and enhances the efficiency and safety of the surgical procedure. Moreover, the Trend Analysis Module enhances the generalization capability of the system’s automatic recognition algorithm, thereby improving the reliability of data used in clinical decision-making. As a result, it effectively supports higher-quality treatment and better clinical outcomes for patients.

## 3 Results

### 3.1 Image segmentation results

To objectively evaluate and compare the segmentation effectiveness of FUAS in the treatment of uterine fibroids, we used the DSC to assess the segmentation quality. The DSC measures the similarity between the automatic segmentation results and the ground truth data, with values closer to 1 indicating higher consistency with the actual boundaries. By adopting this scientific and rigorous evaluation method, we can more accurately assess the performance of FUAS in uterine leiomyoma MRI segmentation. Precision, recall and DSC are represented by Equations 4–6, respectively.

(4) $$\text{Precision}=\frac{\mathrm{TP}}{\mathrm{TP}+\mathrm{FP}}$$

(5) $$\text{recall}=\frac{\mathrm{TP}}{\mathrm{TP}+\mathrm{FN}}$$

(6) $$\begin{array}{c} \mathrm{DSC}=2*\frac{\text{precision}*\text{recall}}{\text{precision}+\text{recall}} \end{array}$$

We will compare and analyze the segmentation results of nnU-Net with those of the HIFUNet model developed by the Southeast University team, as shown in Table 1, nnU-Net significantly outperforms HIFUNet in the segmentation tasks using same data and labels. Additionally, nnU-Net demonstrated excellent performance in segmenting labels not covered by HIFUNet. Based on the comprehensive evaluation by FUAS clinicians, we have determined that the overall segmentation performance of nnU-Net surpasses that of HIFUNet.

**Table 1:** Quantitative comparison of DSC of different segmentation methods on testing dataset.

Labels method	Uterus	Fibroids	Spine	Endometrium	Bladder	Urethral orifice
HIFUNET	83.55%	84.12%	87.33%	83.87%	90.46%	86.76%
nnU-net	87.63%	91.01%	88.98%	87.32%	95.34%	88.11%
Proposed	93.83%	96.27%	93.45%	90.82%	98.37%	91.84%

The DSC of uterus, leiomyoma, spine, endometrium, bladder and urethral orifice segment were 93.83, 96.27, 93.45, 90.82, 98.37 and 91.84%, respectively. These values are detailed in Table 1. Compared with HIFUNet, our approach demonstrates significant advantages in DSC results for uterus, fibroids and spine. Additionally, we are competitive in the endometrial, bladder and meatus segmentation tasks which HIFUNet has not yet explored.

**Figure 14:**
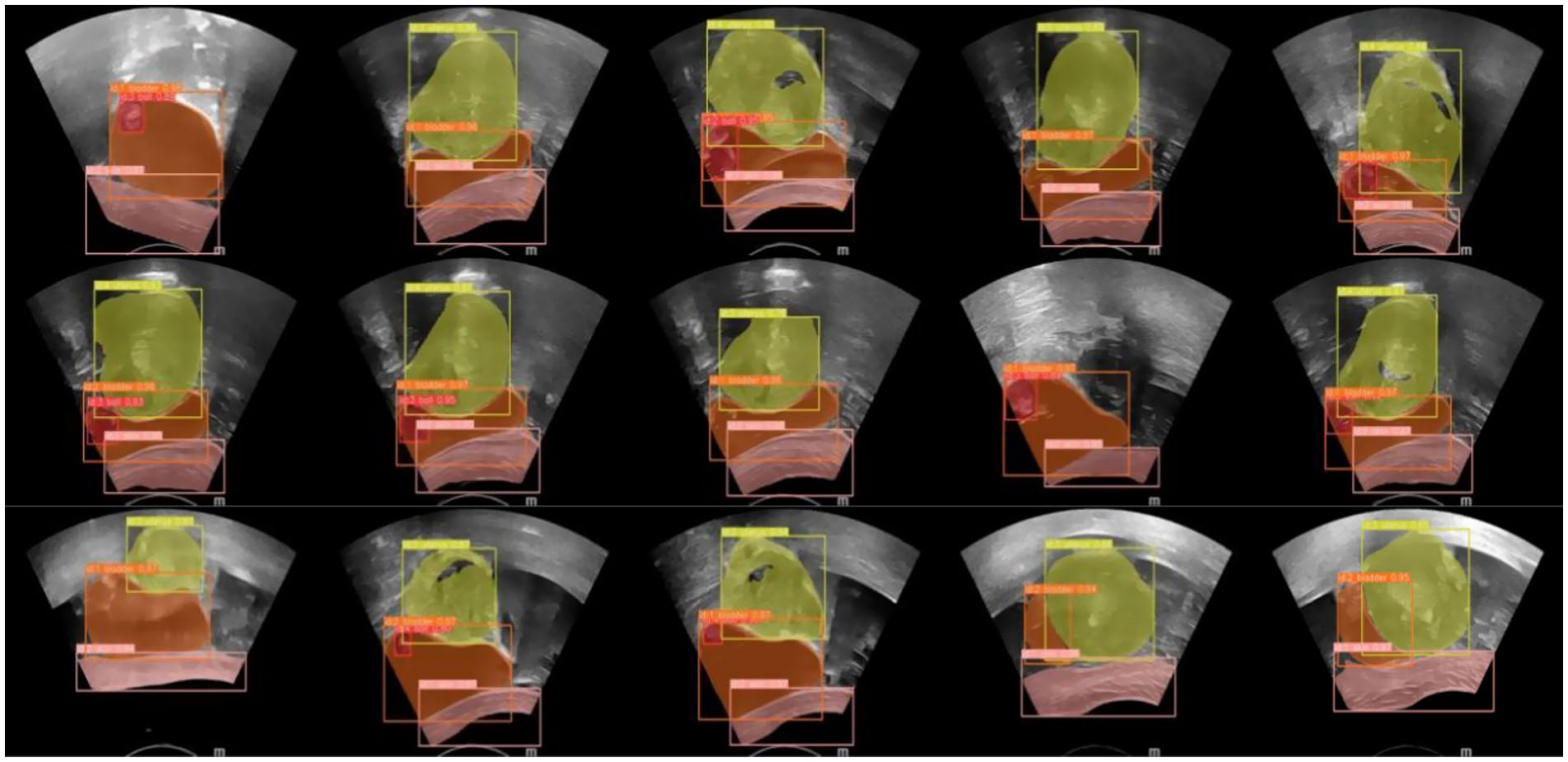
YOLACT Network segmentation results.

An ultrasound image-assisted diagnosis model for FUAS treatment of uterine fibroids was developed based on the YOLACT network. This model assists physicians in preoperative surgical planning and enables accurate intraoperative localization of lesion areas, significantly improving the accuracy of lesion identification and thereby reducing uncertainties caused by human factors. The real-time ultrasound image segmentation model, when combined with MRI segmentation techniques, serves as a valuable auxiliary tool for clinical treatment planning. The model not only maintains high accuracy in clinical real-time ultrasound video diagnosis but also achieves a processing speed of up to 30 frames per second, fully meeting the clinical requirements for real-time ultrasound image segmentation. The segmentation results are shown in Figure 14.

### 3.2 Clinical testing and results

The tracking system has been tested in Chongqing Haifu Hospital, Qinhuangdao Port Hospital and Taiwan Guangtian Hospital, with the results that are generally satisfactory. Table 2 shows the clinical application results from Chongqing Haifu Hospital.

**Table 2:** The clinical application in Chongqing Haifu Hospital.

Type	Patient condition	Evaluation by physicians	Number of cases
Solitary fibroid	The lesion volume is moderate and the image is clear.	Achieve fully automatic registration and localization.	36
	The lesion is relatively large and the images are clear. The morphological matching between MRI and ultrasound is relatively high, but there are certain spatial discrepancies.	The accuracy of automatic registration and localization is relatively high, but the left and right boundaries of the lesion occasionally deviate from the target.	12
Multiple uterine fibroids	Both the lesion and the uterus are relatively large, and the images are clear.	The automatic registration and tracking perform well.	10
	There is a significant angular deviation between the MRI and ultrasound images.	Manual adjustment is required in combination to achieve the target registration and tracking.	50
	The lesions are numerous, large in volume, and widely distributed.	The automatic registration and tracking performance is poor.	2
	Subserosal fibroids, with lesions prone to displacement.	The automatic registration and tracking results are unsatisfactory.	2

**Figure 15:**
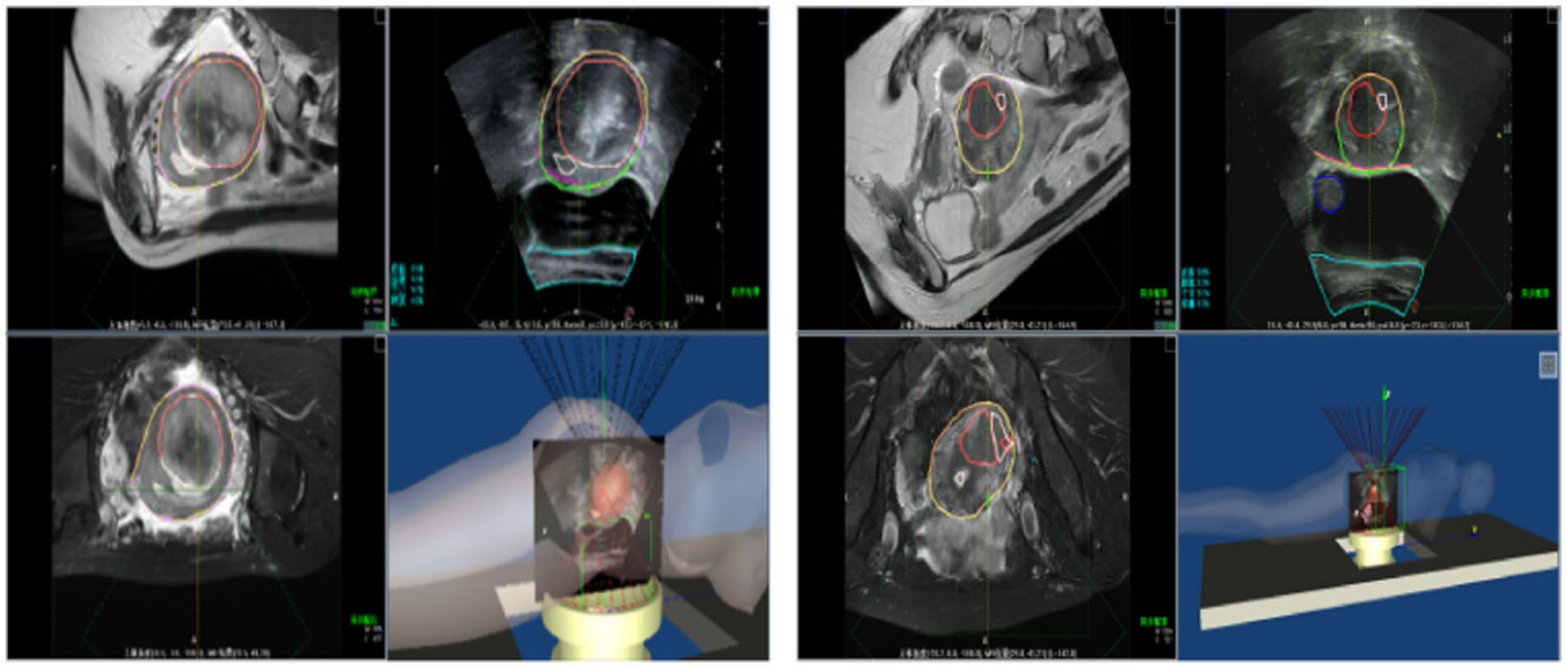
Real-time surgical images tracked the treatment interface.

**Figure 16:**
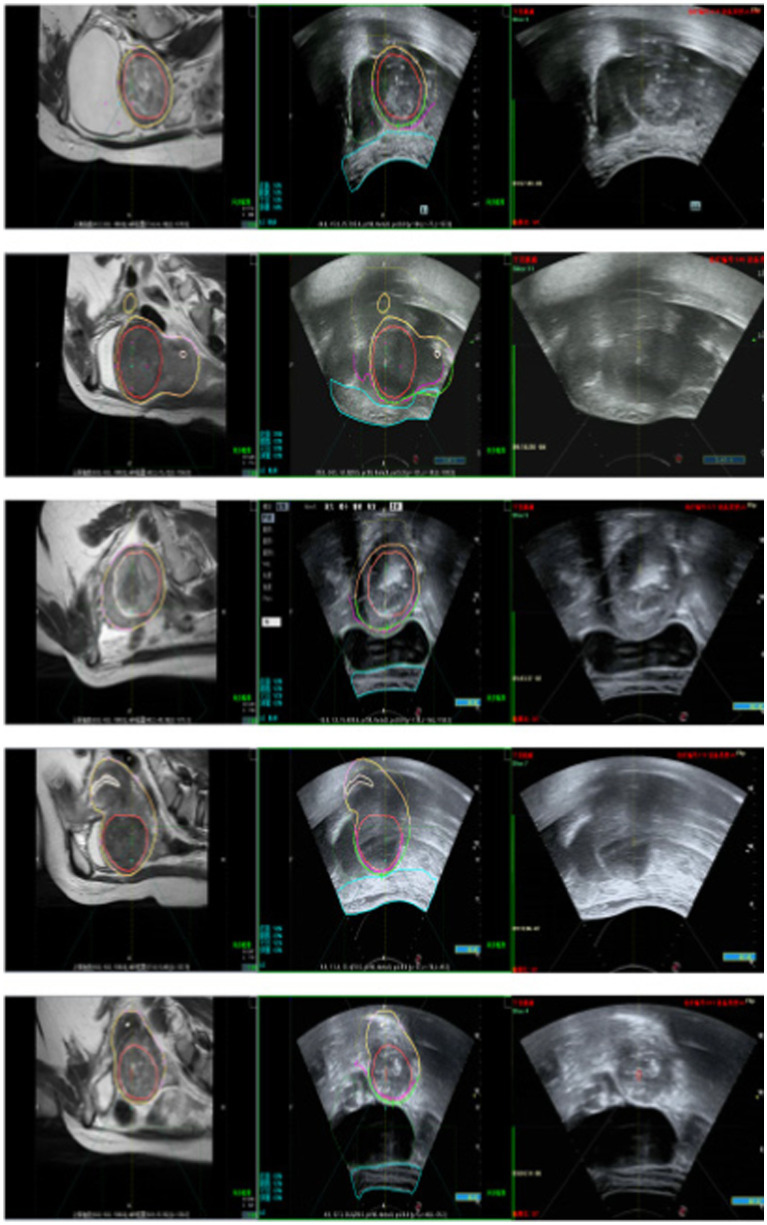
FUAS surgical treatment was followed in real time.

Figure 15 illustrates the tracking of real-time surgical images aligned with the treatment interface, demonstrating the system’s ability to maintain accurate tracking during the procedure. Figure 16 shows the real-time tracking of FUAS treatment, highlighting the effectiveness of the system in providing continuous guidance and monitoring throughout the entire therapeutic process.

**Figure 17:**
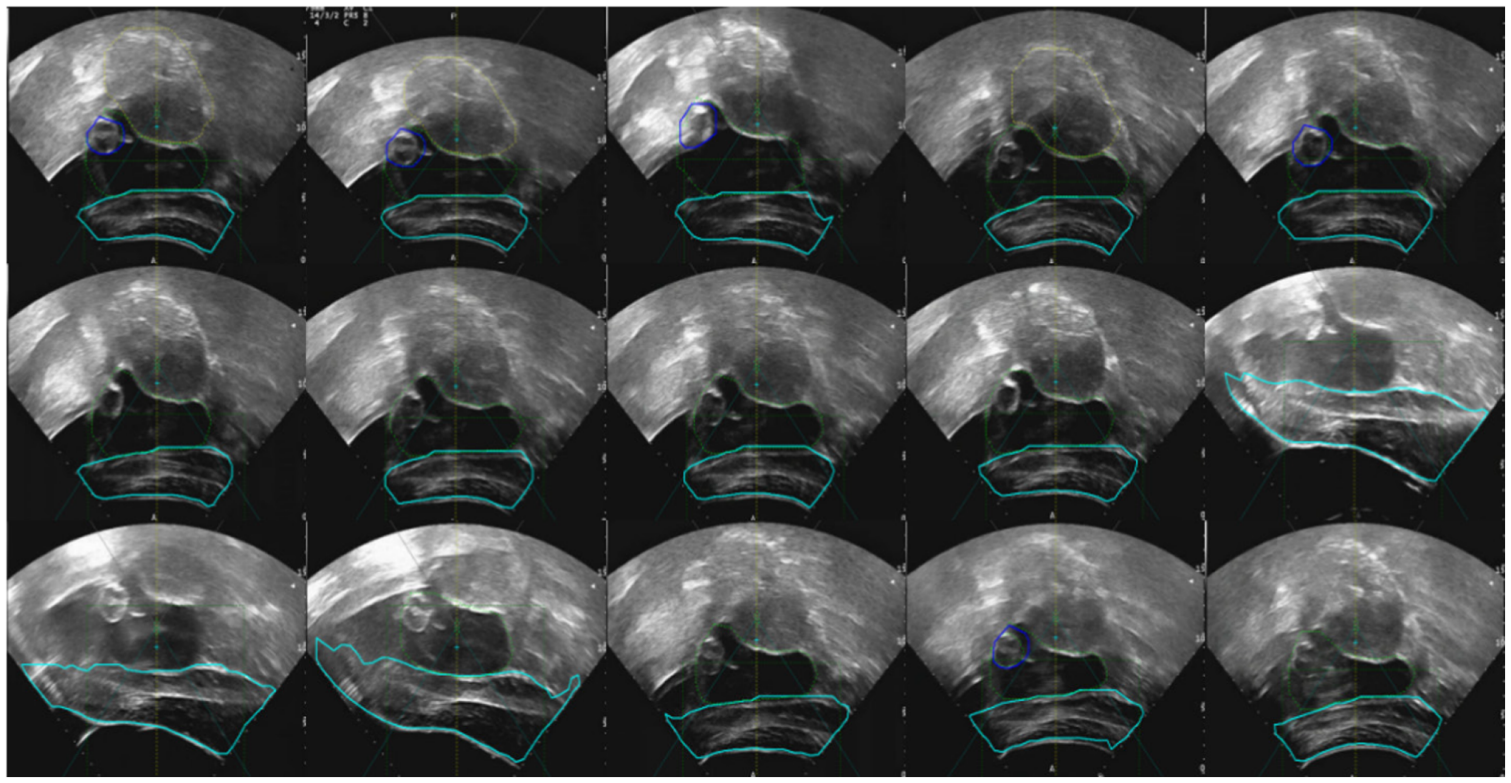
Model generalization ability test.

The tracking and navigation system demonstrated excellent applicability and stability in clinical practice, with its actual performance fully meeting the standards and requirements of surgical procedures. As shown in Figure 17, the system maintained accurate tracking performance even in challenging surgical scenarios involving uterine fibroids with significant deformation. These results fully validate the system’s reliability and efficiency in real-world clinical applications. Its robust performance under various demanding surgical conditions highlights its strong potential for widespread clinical adoption, providing continuous and precise guidance throughout the surgical process. The tracking and navigation system effectively enhances surgical precision and offers a solid foundation for broader clinical implementation and promotion.

## 4 Discussion

FUAS is increasingly favored by both physicians and patients for the treatment of uterine fibroids due to its minimally invasive nature. However, the lack of intuitive 3D visualization during surgery continues to pose challenges for surgical planning and precise targeting. This study enhances the application of FUAS in uterine fibroid treatment by integrating multiple advanced technologies, including nnU-Net, YOLACT, ICP, and trend analysis. Through intelligent processing of preoperative MRI data, a 3D surgical scene is reconstructed to support preoperative planning. In addition, the fusion of preoperative MRI with intraoperative real-time ultrasound images significantly improves the accuracy of fibroid localization.

This study addresses two key issues: (1) Achieving intelligent 3D scene visualization based on MRI to assist in preoperative planning; (2) Proposing a multimodal image registration method for intraoperative FUAS navigation based on the recognition of local features in real-time ultrasound images.

The proposed method not only enhances the accuracy of preoperative localization but also enables continuous intraoperative tracking of uterine fibroids, effectively addressing the challenges of 3D visualization and navigation in minimally invasive surgery and greatly improving clinical efficiency. The integration of these technologies simplifies the clinical workflow and elevates the level of surgical intelligence and automation. In approximately 90% of cases, the method demonstrated excellent tracking performance, comparable to manual registration by experienced clinicians. Nonetheless, this study has several limitations, including its single-center design, a relatively small sample size, and potential subjectivity in clinical assessments. The system’s tracking performance also declines to some extent in cases involving large-scale tissue deformation.

When the position or shape of the uterine fibroid changes significantly, such as due to the patient’s posture or large morphological changes in the fibroid itself, these deformations lead to inconsistencies in shape and structure between images, reducing the accuracy of registration and target tracking. In the future, a deeper analysis of tissue deformation patterns will be conducted, and real-time tracking techniques, such as motion tracking algorithms, will be employed to maintain image consistency and improve the system’s accuracy under large deformations. Furthermore, further optimization of the fusion between preoperative MRI and intraoperative ultrasound images will provide doctors with more comprehensive lesion information, allowing for the development of more scientifically-based and efficient FUAS treatment plans, thus shortening the learning curve and reducing localization time.

## 5 Conclusion

In this study, the preoperative MRI images were first segmented and reconstructed in 3D using the nnU-Net framework, enabling detailed visualization of the morphology of uterine fibroids and their spatial relationships with adjacent anatomical structures to support surgical planning. Subsequently, the YOLACT network was employed for rapid identification of the uterus and other critical anatomical structures. Finally, the ICP algorithm and trend analysis method were introduced to register preoperative MRI with intraoperative ultrasound images, thereby enhancing the accuracy of lesion localization and navigation during FUAS procedures. The proposed approach achieves automatic segmentation, fused navigation, and dynamic tracking of lesion areas in both MRI and ultrasound images, demonstrating strong practical applicability. This method effectively reduces the operational burden on clinicians and improves the intelligence and automation of the surgical workflow. By integrating nnU-Net, YOLACT, and ICP techniques, the proposed system successfully addresses the challenges of 3D visualization and navigation in minimally invasive uterine surgeries, showing particularly strong performance in cases with minimal lesion deformation.

## Data Availability

The data analyzed in this study is subject to the following licenses/restrictions: the dataset used in this study contains medical imaging data (MRI and ultrasound) from 638 patients collected at Chongqing Haifu Hospital between January 2021 and January 2023. Due to ethical and legal considerations, including patient confidentiality and data protection regulations, the dataset cannot be publicly shared. Access to the raw data may be granted upon reasonable request and with appropriate institutional approvals, subject to compliance with ethical guidelines and data-sharing agreements. Requests to access these datasets should be directed to 29295778@qq.com.
